# Choosing between nurse-led and medical doctor-led from private for-profit versus non-for-profit health facilities: A household survey in urban Burkina Faso

**DOI:** 10.1371/journal.pone.0200233

**Published:** 2018-07-25

**Authors:** Idrissa Beogo, Amadou Darboe, Oluwafunmilade A. Adesanya, Bomar Mendez Rojas

**Affiliations:** 1 Centre de recherche en gestion des services de santé, FSA-ULaval-CHU de Québec UL–IUCPQ-UL, Pavillon Palasis-Prince, Université Laval, Québec (Qc), Canada; 2 École Nationale de Santé Publique, Ouagadougou, Ouagadougou, Burkina Faso; 3 The University of Melbourne, School of Population and Global Health, Parkville, Victoria, Australia; 4 Institute of Public Health, International Health Program, National Yang Ming University, Beitou District, Taipei, Taiwan; Makerere University, UGANDA

## Abstract

**Background:**

Providers’ qualification (Medical doctor [MD] or nurse); type of care facility ownership (for-profit [FP] or not-for-profit [NFP]) may all influence individuals’ healthcare-seeking behavior and therefore merits empirical assessment to provide valuable evidence-informed policy orientation in the present context of private health system development. Previous studies have not examined these factors in combination, especially within the urban context of sub-Sahara Africa, where the private sector is rapidly growing. This study aims to explore factors associated with urban residents’ preferences between private MD-led and private nurse-led outpatient care and how these factors vary by type of private health facility ownership (FP and NFP) and levels of disease severity (severe and non-severe cases).

**Methods:**

A cross-sectional household survey was conducted in July-November 2011 on a random final sample of 2064 adults (646 households). We used a face-to-face interview to capture participants’ choice of provider and their associated factors. A multivariable logistic regression was applied.

**Results:**

For severe conditions, participants, almost equally sought FP and NFP facilities, only 36.4% preferred nurses compared to MDs, while for non-severe cases 53.2% preferred FP facilities and only 29.2% patronized nurses. For non-severe conditions, university educated were more likely to use MDs-led FP compared to nurse-led FP facilities (Odds Ratio [OR] = 4.66, 95% confidence interval [CI] = 2.62–8.30) and MD-led FP over MD-led NFP facilities (OR = 1.03, 95%CI = 1.01–1.04), for severe health conditions. Having insurance predicted MD-led FP preference over nurse-led FP. Furthermore, insurance predicted the preference for MD-led FP over MD-led NFP facilities. Employment did not distinguish participants’ choice of provider.

**Conclusion:**

The findings suggest that, at different levels, MDs and nurses from FP and NFP facilities importantly contribute to health services delivery regardless of the severity of health conditions. The results offer some valuable evidence for policy orientation in the current rising tide of the private system, including workforce development, and practitioners' role definition. We suggested that health insurance mechanism would reinforce the private health services utilization and could enhance progress towards the attainment of Sustainable Development Goals.

## Background

Recent publications have increasingly paid attention to the mounting issue of private besides government-owned health sector—traditionally entirely state-run [1]. In the last two decades, middle- and low-income countries (LMICs) have been experiencing increasing privatization of healthcare [2–5], a phenomenon which might be induced by the rapid globalization and the adoption of the free-market policy in many countries since the 1990s. The growing privatization of healthcare is impacting healthcare delivery, financing, and stewardship as well as the pattern of care-seeking behavior especially in urban areas [6]. Additionally, it has given rise to a variety of provider ownership, including, for instance, public, private for-profit (FP) and private not-for-profit (NFP) ownership.

Healthcare systems in many sub-Sahara African (SSA) countries have been structured since the colonial period and they resemble much of the colonizers’ system. Before and after the World War I, the colonial masters recruited and trained African nurses to struggle on the forefront against grand endemics. Their role has remained prominent over the successive reforms and their contribution is considered pivotal in providing primary care in many SSA countries.

In Burkina Faso for instance, reliance on nurses started since the 1960s and remains a means of alleviating the hindrance of protracted physician shortage [7]. Nurses play a critical role in primary health care, providing essential health services to individuals and families [8], which eventually enhances more equitable access to basic healthcare services in the country, as acknowledged by the World Health Organization (WHO). Thus, based on the premise that nurses have to be competent to practice with the autonomy of diagnosis and treatment decision under minimal supervision, the WHO has developed nurses training guideline for its member countries in SSA [9].

Contrary to Burkina Faso which is a low-income country, in high-income countries (HICs) such as Australia, Canada, US, and the UK, physicians shortage and cost constrain later on have been the leading raison d’être of physician assistant (PA) and nurse practitioner (NP) emergence [7,10]. In the US for instance, both the NP and PA professions began in 1965. PAs education at Duke University framed on a fast-track doctor curriculum while NPs program was developed and implemented at the University of Colorado [7]. The US success story prompted their recent development and implementation in the UK, New Zealand, Australia and Ireland [11].

Once physician assistants [12,13], nurses have increasingly broadened their practice competence and upgrade their autonomy through specialized practices which allows them to overlap and even encompass physician traditional prerogatives [14]. Nurses are increasingly capable of attending to different patient groups [11,15] which demonstrates their broad clinical knowledge and skills. A number of studies suggest that nurses proved to be as competent or even more than doctors at primary healthcare level [16,17]. The first-ever meeting of the World Health Assembly ([WHA] 1.46, 1948) passed a resolution that recognized the role of nurses in primary health care delivery [18]. The 2005 stakeholders meeting and many that followed it [i.e. sixty-fourth WHA [19]] re-acknowledged their crucial contribution to the ultimate achievement of the Millennium Development Goals (MDGs) [8]. Nursing curricula in SSA are tailor-made to the context of boosting primary care promotion. Like other SSA countries, in Burkina Faso, nurses and midwives spearhead primary healthcare provision, thereby contributing towards the achievement of the country’s Sustainable Development Goals (SDGs).

The growing enthusiasm in SSA for private sector intervention in addressing some of the conundrums in the healthcare industry, fueled by the free market ideology [20] and other domestic contingencies (democratization waves, public budget strain, etc.) significantly bolstered health systems’ performance [3] and generated an array of private healthcare providers. Given its breadth, scope, and size, the engagement of the private sector is regarded as an alternative to complement the public sector and it has been encouraged for a more significant role toward achievement of MDGs achievement [21]. In Burkina Faso, nurses and midwives have embarked on private practices venture following the market liberalization policy in 1991, thus, competing in the market with MDs.

The traditional literature has mainly paid attention to MDs’ practice. Their services are considered the benchmark for measuring appropriateness and quality of clinical care; however, are inapposite in measuring interpersonal quality or art of care, which is the essence of nursing [22]. With the exception of some HICs (e.g. USA, United Kingdom), to date, there has been limited literature [23] on the direct comparison between MDs and nurses in LMICs. Further, the private sector is characterized by poor regulation and scanty information [24] and ineffective governmental control [25]. Hitherto, very few studies have investigated private healthcare practices to identify the roles of different components of in a private system (including FP versus NFP).

To our knowledge, this study is one of the first to explore such a topical issue in the shared-care market between nurses and physicians in the context of the private healthcare sector in a resource-limited setting. Further, it is among the first to directly compare preferences for MD and nurse services while putting into account the type of health facility ownership and varied health conditions (severe and non-severe). Based on the scarce existing literature and the study background private health operating system, we hypothesized that patient choice of healthcare provider would not significantly differ between nurses and MDs regardless of the type of ownership.

### Healthcare operating system

Following the waves of economic liberalization and nascent democratization in SSA in the early 1990s, the private sector has gained impetus. Burkina Faso has experienced a rapid growth resulting today with the highest concentration of private healthcare providers, 65% in the capital city, Ouagadougou. The private sector consists of FP and NFP health facilities. The FP facilities, established since the early post-colonial era [26], have been exempted from public stewardship and have the ultimate aim of making a profit. NFP facilities are from charitable obedience and their initiation date back to the pre-colonial era. Initially from Christian religion (and Islam later on) [27,28]; they now include, civil society organizations, domestic and international faith- and non-faith-based organizations. Nevertheless, they still benefit from a long-lasting philanthropic support associated with charitable organizations e.g., the Catholic religious sisters’ services ―still present― that are merciful, fraught with patience, and relatively cheap. They adhere to government policies, deliver similar programs like government-owned (public) facilities (eg., health promotion, preventive, and curative services…). Contrary to FP, in NFP setting the freedom to directly meet an MD of one's choice is limited.

Structurally, private sector system in Burkina Faso is unique, unfitting the traditional three-tier hierarchical healthcare delivery policy. Unlike some of the LMICs [25], the private health sector of Burkina Faso (with the exception of NFP facilities), is very less involved in the delivery of some of the public health programs (e.g., immunization, child and maternal services, TB, HIV/AID…) which are often free services. Healthcare is sought basically on a walk-in, without a priori gatekeeping mechanism, and services are overwhelmingly paid on out-of-pocket [29,30]. Both NFP and FP differ considerably, ranging from small businesses in crudely arranged offices to high-tech and well-equipped hospitals. NFP and especially most of the FP MD-led providers operate by hiring moonlighting qualified professionals and medical students.

## Methods

### Setting

In planning the implementation of this population-based study, it was important that: 1) the study targeted an area that is broadly a typical urban setting and 2) served by a representative network of different provider sponsorships: non-conventional (e.g., traditional healers, marabouts), public and private (NFP and FP). Based on the available statistics, we decided to select the capital city, Ouagadougou. It has the most heterogeneous population and the highest concentration of facility networks in the country, including primary care centers (about 60), district health centers (n = 5) and University hospitals (n = 3) [31]. The latter serve as national referral hospitals. By the study period, there were 49 FP and 23 NFP MD led facilities. Additionally, there were 118 nurse-led facilities (including, n = 8 midwife facilities), and 13 primary health centers mainly of NFP.

### Sampling and population

The sample was derived from a large project on "Healthcare-seeking behavior in an urban area", that covered 1,600 households in Ouagadougou. This initial project applied a two-stage random sampling technique, details of which has been described elsewhere [6]. The data were collected between July and November 2011, from households established at least six months prior to the survey. The sample for the present study (due to our research questions) was restricted to 2,411 adults (from 646 households) living in the metropolitan area of Ouagadougou, who identified a private healthcare provider (MD or nurse) as their usual source of care. Of the selected sample, a total of 2,064 adults (86%) had complete information and thus, constituted the final analytical sample. Data were collect through face-to-face interviews using semi-structured questionnaires. Quality control mechanisms were employed to ensure accuracy in the collection and processing of survey data. In a case, for instance, where a respondent failed to clarify facility ownership and/or provider qualification, the research team would conduct a site visit and check facility roster to confirm. This measure aimed to minimize misclassification. Household heads (usually men) and their spouses—entirely females—(if any) were separately surveyed and the spousal (females’) responses were retained, assuming they know the household disease event better than men. Six interviewers were selected and trained. The questionnaire was pilot-tested on 32 households and the principal investigator (IB) has supervised 12% of the clusters selected and repeated two percent of the interviews using a short version of the questionnaire with unalterable (fixed) variables. Finally, a number of data checks were run to flag out errors including for example; checking qualities of responses on questionnaires and double-checking data when entering into the CSPro version 4.

### Data and variable description

For health condition related information, two types were defined, severe and non-severe cases, and data were collected on all the adults of selected households. This study considers visits to a private healthcare provider as the first outcome measure and the practitioner qualification, the second. Thus, only participants who choose private providers were retained in the analytic sample. Private providers are defined here as "organizations working outside the direct control of the State” [32], and are further classified into MD-led ―facilities managed by a physician― and nurse-led ―facilities run by a registered nurse (RN) or a nurse practitioners (NP) (regardless of the specialty) or registered midwives. With regards to the type of facility ownership, providers were categorized into FP or NFP.

Explanatory variables included gender, age, insurance, education (no education, primary, junior, junior high, and university), occupation (public & para-public workers, formal private, informal private, not in labour (retirees & household wives, students and jobless), marital status (married, single and other), and health condition (severe and non-severe). More specifically, we defined a severe condition as a medical condition with a perception of fatality in the absence of an urgent intervention. The following common symptoms were used as illustrative examples: loss of consciousness, coma (generally in a context of a fever), tachypnea, a fracture, or a bad injury caused by an accident. Non-severe conditions were defined by the interviewer as a condition that may compromise one daily life activities and/or workability in the absence of a medical care. Mild headaches, stomachaches, fevers, shivers, and coughs were some symptoms used for illustration.

### Statistical analysis

Descriptive statistics of the sample characteristics included frequencies and proportions of all variables for the two conditions of interest (severe and non-severe). Owing to dichotomous measures, multivariable logistic regression models for the likelihood of the primary care visit being attended by a nurse compared to a doctor was used for each of the conditions to figure out respondents’ choice of a care provider. Odds ratios (ORs) and 95% confidence intervals (CIs) were calculated. Based on the study hypothesis, the models applied have been compared separately by both assessed conditions, with the following scenario: 1) different qualifications (MD against nurse) from same ownership and 2) providers of the same qualification but from different ownerships (FPMD versus NFPMD and FP nurse versus NFP nurse). 3) Finally, providers were pooled and assessed by ownership (FPMD and FP nurse versus NFPMD and NFP nurse). Statistical significance was defined as a 2-tailed *p* < 0.05 and all analyses were performed using SPSS version 21 (SPSS inc., IL: Chicago, USA, 2009).

### Ethics considerations

We interviewed only household heads and their spouses who answered the questions for themselves and for all other household members. The informed consent statement was presented on the front page of the questionnaire. This was read and explained to each individual respondent and ended by their response labeled by a “yes” for agreeing or “no” for declining to participate. Instead of a written consent, the oral consent sought complies with the standard protocol of the Burkina Faso national demographic and household surveys. The study was approved by the Burkina Faso National Ethics Committee for Research (#2011-11-82, 11 November 2011). An administrative permission was further obtained from the Ouagadougou Town Council.

## Results

### Baseline characteristics

**Fig 1:**
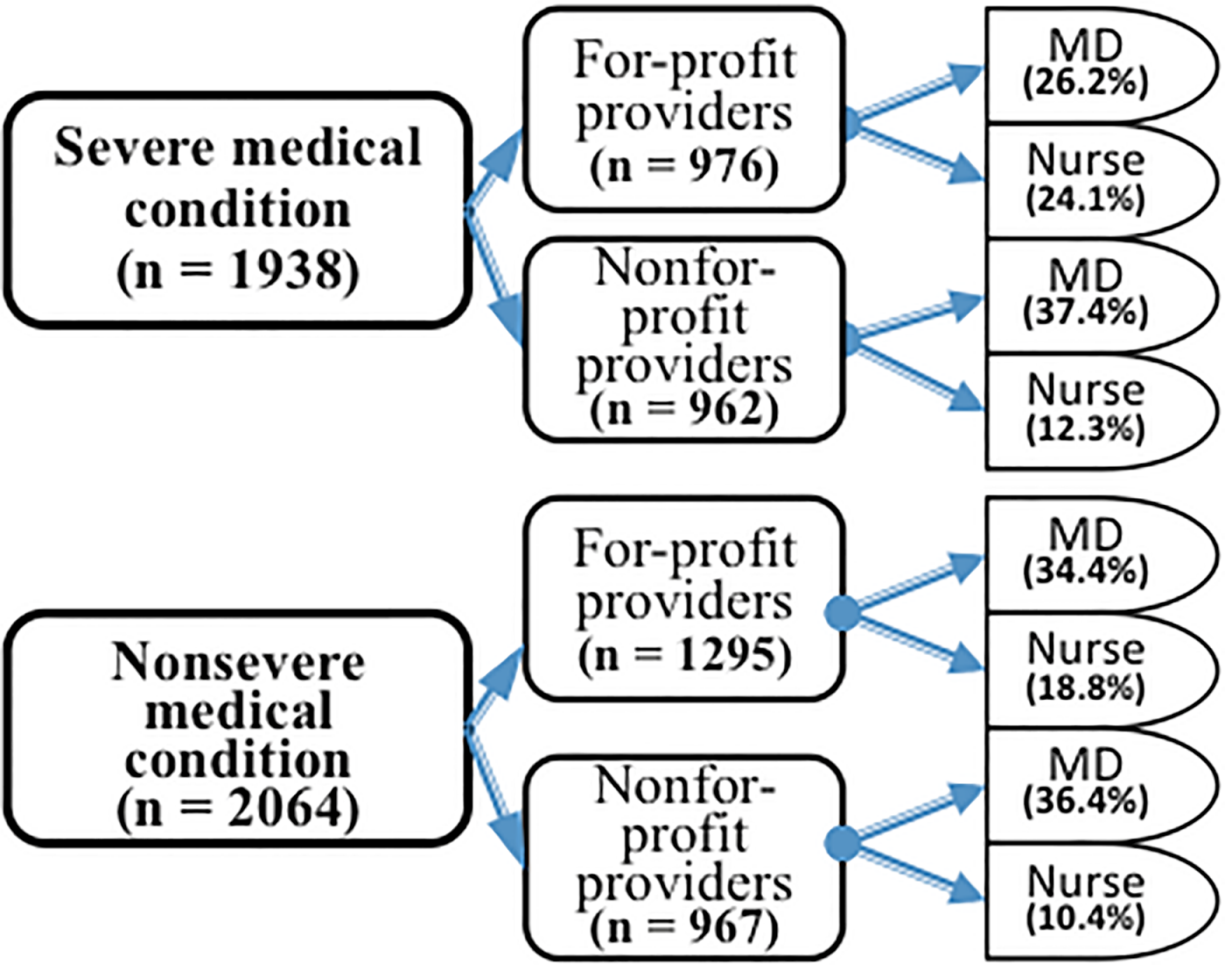
Private healthcare providers sought by qualification and ownership.

In total, 646 households were surveyed, that finally included 2064 individuals. For severe health conditions, participants almost equally sought FP (n = 976) and NFP (n = 962) facilities and notably, only 36.4% (n = 706) preferred nurses over MDs. For non-severe conditions, 53.1% (n = 1295) preferred FP facilities and in terms of qualification, 29.2% (n = 603) favored nurses (**Fig 1**). The mean age of the study participants was 33.2 (SD 14.7) years; of whom, 51.7% were women, 51.0% married or in union, 35.3% attended junior education or higher. About 4.1% were insured and 42.8% were gainfully employed **(Table 1).** Of the surveyed participants, a total of 1,232 (NFP: n = 724, FP: n = 508) and 706 (NFP: n = 238, FP: n = 468) would go to MD and nurse-led facilities for severe conditions respectively. For non-severe conditions, 1,461 (NFP: n = 752, FP: n = 709) would visit MD-led facilities whereas 603 (NFP: n = 215, FP: n = 388) would go to nurse-led facilities.

**Table 1:** Descriptive characteristics of the study sample (n = 2064).

	n	%
**Age**, M(SD)	33.2 (14.7)	
**Gender**		
Female	1067	51.7
Male	997	48.3
**Marital status**		
Married & free union	1052	51.0
Single	922	44.7
Divorced-separated	90	4.4
**Education**		
University (grade ≥14)	313	15.2
Junior high (grade 11–13)	414	20.1
Junior (grade 7–10)	561	27.2
Primary (grade 3–6)	285	13.8
No education	491	23.8
**Occupation**		
Public & parapublic worker	274	13.3
Formal private	136	6.6
Informal private	473	22.9
Not in labour (retirees & household wife	465	22.5
Student	579	28.1
Jobless & others	137	6.6
**Insurance**		
Insured	84	4.1
Uninsured	1980	95.9

### Multivariate analyses

In **Table 2**, the multivariable logistic regression compares qualification levels across different facility ownership. Across health conditions, age showed a significant difference in the likelihood of choosing FP MD over nurse for non-severe conditions (OR = 1.01, 95% CI = 1.00–1.03). No significant difference was found for sex across presenting conditions, however, participants' education exhibits a significant difference for both qualifications and ownership, with a dose-response relationship. Thus, for severe conditions, university educated participants have almost two folds likelihood of choosing MD-led FP facilities over nurse-led FP facilities (OR = 4.39, 95% CI = 2.48–7.77) versus (OR = 2.36, 95% CI = 1.50–3.71) for participants with junior education level. A similar pattern was found between MD-led NFP versus nurse-led NFP. For non-severe medical conditions, the odds of seeking MD-led FP versus nurse-led FP facilities is 2.7 times higher for those who attended university level compared to junior level. In the same comparative scheme, the OR is 3.7 times higher when comparing MD-led NFP to nurse-led NFP facilities. Finally, there is no significant difference shown for occupation, but insured participants exhibit a greater likelihood of seeking MD-led FP facilities for severe (OR = 3.01, 95% CI = 1.46–6.17) and non-severe conditions (OR = 2.59, 95% CI = 1.28–5.23).

**Table 2:** Multivariable logistic regression of private healthcare utilization by severity of condition and providers’ qualification (MD and nurse).

	Severe				Non-severe			
	FP MD-FP nurse		NFP MD-NFP nurse		FP MD-FP nurse		NFP MD-NFP nurse	
	AOR	95% CI	AOR	95% CI	AOR	95% CI	AOR	95% CI
**Age**, year	1.00	0.99–1.02	1.00	0.99–1.01	1.01	1.00–1.03	1.01	0.99–1.03
**Gender**								
Male	1		1		1		1	
Female	1.17	0.86–1.58	1.03	0.73–1.46	1.30	0.96–1.74	0.98	0.68–1.41
**Marital status**								
Divorced	1		1		1		1	
Married & free union	0.72	0.35–1.48	0.84	0.41–1.71	0.95	0.44–2.04	0.79	0.35–1.78
Single	0.62	0.26–1.49	0.57	0.24–1.36	1.16	0.47–2.83	0.64	0.24–1.68
**Education**								
No education	1		1		1		1	
University	4.39	2.48–7.77	2.97	1.42–6.23	4.60	2.63–8.05	8.98	3.50–23.04
Junior high	3.28	1.95–5.51	2.74	1.51–4.98	2.17	1.33–3.53	3.33	1.78–6.24
Junior	2.36	1.50–3.71	1.75	1.12–2.74	1.70	1.12–2.60	2.43	1.51–3.93
Primary	0.88	0.54–1.43	0.98	0.61–1.57	0.70	0.44–1.10	1.88	1.16–3.06
**Occupation**								
Jobless	1		1		1		1	
Public & parapublic	0.82	0.38–1.76	0.57	0.22–1.47	0.82	0.38–1.76	0.34	0.12–0.95
Formal private	0.83	0.36–1.91	0.76	0.27–2.16	0.78	0.34–1.77	0.17	0.06–0.48
Informal private	0.55	0.28–1.05	0.46	0.23–0.93	0.63	0.33–1.20	0.23	0.11–0.46
Retired & HH wife	0.69	0.35–1.37	0.61	0.29–1.29	0.67	0.34–1.32	0.43	0.20–0.93
Student	0.58	0.30–1.14	0.66	0.31–1.39	0.79	0.40–1.54	0.37	0.17–0.81
**Insurance**								
No	1		1		1		1	
Yes	3.01	1.46–6.17	1.11	0.31–4.02	2.59	1.28–5.23	0.55	0.17–1.83

AOR, Adjusted odd ratio; CI, confidence interval; FP, for-profit; NFP, not-for-profit; MD, medical doctor.

**Table 3** presents the likelihood of choosing a provider with same qualification but from different facility ownership. There appeared to be a positive correlation in the likelihood of visiting MD-led FP facilities over the MD-led NFP ones. For severe and non-severe conditions alike, age appeared to have a positive association with choices for both MD- and nurse-led FP facilities. For both assessed conditions, females tend to recourse to MD-led FP facilities, while, the single would likely go to FP facilities disregarding provider qualification. Furthermore, it appeared that the higher people are educated, the more they are likely to choose MD-led FP facilities regardless of the severity of their health condition. The odds is 2.84 (95%CI = 1.71–4.70) and 2.26 (95%CI = 1.45–3.50) for severe and non-severe conditions, respectively, for participants who attended a university level. Although public, para-public and formal private jobholders are likely to use MD-led FP facilities; there is no statistical association for severe cases. It however, appeared that for non-severe conditions, these groups would prefer MD-led FP facilities. Finally, people with an insurance plan were significantly more likely to seek an MD-led FP facility—only—in case of a severe health condition (OR = 3.58, 95% CI = 1.97–6.52) or non-severe medical condition (OR = 4.57, 95% CI = 2.40–8.71).

**Table 3:** Multivariable logistic regression of the choice of providers with same qualification but from different ownership by severity of condition.

	Severe				Non-severe			
	FP-MD vs NFP-MD		FP-Nurse vs NFP Nurse		FP-MD vs NFP-MD		FP Nurse vs NFP Nurse	
	AOR	95% CI	AOR	95% CI	AOR	95% CI	AOR	95% CI
**Age**, M(SD)	1.03	1.01–1.04	1.02	1.00–1.04	1.02	1.01–1.03	1.02	1.00–1.04
**Gender**								
Male	1		1		1		1	
Female	1.56	1.19–2.04	1.40	0.96–2.02	1.47	1.15–1.87	1.37	0.915–2.05
**Marital**								
Divorced	1		1		1		1	
Married & free union	1.55	0.84–2.83	1.77	0.78–4.03	1.98	1.10–3.57	1.52	0.59–3.94
Single	2.32	1.11–4.84	2.32	0.86–6.27	3.16	1.56–6.41	1.49	0.49–4.55
**Education**								
No education	1		1		1			
university	2.84	1.71–4.70	1.76	0.81–3.84	2.26	1.45–3.50	4.03	1.47–11.05
Junior high	2.22	1.40–3.51	1.66	0.86–3.21	1.61	1.07–2.43	2.38	1.20–4.72
Junior	2.20	1.45–3.31	1.46	0.89–2.39	1.56	1.10–2.23	2.16	1.26–3.71
Primary	1.64	1.00–2.67	1.77	1.09–2.86	0.92	0.60–1.39	2.34	1.39–3.95
Occupation								
Jobless	1		1		1		1	
Public & parapublic	1.46	0.75–2.83	1.01	0.36–2.77	2.24	1.24–4.05	1.05	0.34–3.24
Formal private	1.51	0.74–3.10	1.03	0.34–3.15	3.34	1.71–6.53	0.65	0.22–1.93
Informal private	0.80	0.44–1.44	0.59	0.28–1.24	1.63	0.98–2.73	0.56	0.25–1.26
Retired & HH wife	0.96	0.52–1.75	0.77	0.34–1.73	1.47	0.86–2.51	0.79	0.33–1.93
Student	1.20	0.68–2.14	1.12	0.50–2.53	2.27	1.36–3.79	1.06	0.43–2.61
Insurance								
No	1		1		1		1	
Yes	3.58	1.97–6.52	1.34	0.35–5.13	4.57	2.40–8.71	0.83	0.24–2.87

AOR, Adjusted odd ratio; CI, confidence interval; FP, for-profit; NFP, not-for-profit; MD, medical doctor.

**In Table 4**, nurses- and MD-led facilities were pooled into their ownership types (FP and NFP). Thus, the multivariable logistic regression exhibits the likelihood of choosing FP facilities over NFP ones. In both medical conditions, increase in age, females and insurance holders were more likely to go to FP nurses- and MDs-led than NFP ones. As for education, although highly educated markedly preferred FP, there was no dose-response pattern. Similarly, for occupation, higher paid jobholders (formal private) chose FP facilities for severe (OR = 1.72, 95%CI = 0.91–3.23) and non-severe (OR = 2.43, 95%CI = 1.40–4.20), but least paid categories also recourse to FP.

**Table 4:** Multivariable logistic regression of the choice of FP versus NFP health facilities, by severity of condition.

	FP-NFP Severe condition		FP-NFP Non-severe condition	
	AOR	95% CI	AOR	95% CI
**Age**, M(SD)	1.03	1.01–1.04	1.02	1.01–1.03
**Gender**				
Male	1		1	
Female	1.49	1.18–1.88	1.38	1.13–1.69
**Marital**				
Divorced	1		1	
Married & free union	2.01	1.16–3.49	1.91	1.17–3.10
Single	3.31	1.72–6.37	2.46	1.38–4.39
**Education**				
No education	1		1	
University	1.74	1.13–2.68	1.99	1.36–2.91
Junior high	1.56	1.06–2.29	1.50	1.07–2.11
Junior	1.61	1.15–2.25	1.51	1.13–2.01
Primary	1.92	1.34–2.75	1.29	0.95–1.76
**Occupation**				
Jobless	1		1	
Public & parapublic	1.40	0.78–2.48	1.91	1.16–3.14
Formal private	1.72	0.91–3.23	2.43	1.40–4.20
Informal private	0.77	0.48–1.24	1.39	0.92–2.09
Retired & household wife	0.91	0.55–1.50	1.38	0.89–2.12
Student	1.24	0.75–2.04	2.11	1.37–3.22
**Insurance**				
No	1		1	
Yes	2.86	1.60–5.10	3.19	1.82–5.59

In the models run, both FP and NFP included the pool of nurse- and MD-led facilities.

AOR, Adjusted odd ratio; FP, for-profit; NFP, not-for-profit; MD, medical doctor.

## Discussion

This study is among the first of its kind to compare health care utilization putting into account individual choices between nurse- and MD-led outpatient cares in private clinical settings. Specifically, we examined factors driving these individual choices in the context of urban Burkina Faso. Essentially, provider qualification (nurse or MD) and health facility ownership types (FP and NFP) were assessed. This study becomes necessary owing to the rapid development of private healthcare sector in SSA since the endorsement of free market policy. Hence its findings could be valuable for policymakers. The present study laid out four salient findings.

First, for both assessed medical conditions (severe and non-severe), the results laid out that, on one hand, highly educated people and insurance holders were more likely to choose an MD over the nurse. On the other hand, MD led FP or nurse-led FP facilities were more preferred in case of severe health conditions only. Indeed, important literature did compare doctors to nurse practitioners (NPs) and/or physician assistants (PAs) in clinical practice, however; few have made the comparison in light of the severity of health conditions or the type of health facility ownership. The United States is home to the majority of the published studies. Overall, a wealth of existing literature concurred on similar findings. For Everett et al. [33], predisposing and enabling factors (gender, age, metropolitan residence, or uninsured or on public insurance) were associated with identification of PA/NPs as a main source of care. For others, the drivers are related to efficiency (cost/quality) [34], or equal performance with MD in the primary care service delivery, as reported by Arts et al. in their randomized control trial (RTCs) [35] and Martin-Misener et al.'s systematic review [36], or significantly higher level of trust of the patients [37]. For Matteliano and Street [17], what differentiates nurses was the comprehensiveness of their cultural competence approaches, while Dierick-van et al [38] argued that NPs emphasized on their patient's follow-up consultations, which were significantly longer. Finally, Bodenheimer's [39] landmark study has proposed RNs as a solution to fill the primary care MD shortage, as they perform equally or better.

The second salient finding indicated that apart from comparing pooled providers (FP vs NFP), participants' occupation did not significantly differentiate their choices between the MD and nurse-led care. This appeared to contradict the findings of Das & Das [40], for whom the degree of wealth is proportional to the qualification of the provider resorted. In other words, those in the higher wealth quintiles may prefer to be attended to by MDs, who are theoretically more qualified and equipped than nurses. Nevertheless, our finding concurred with what has been largely reported in the mainstream literature. For primary care, nurses performed equally better in comparison to MDs [16,41–43], receive more satisfaction from patients [44,45] and exercise superior interpersonal skills [12,44]. In our study, the majority of private nurse-led facilities are managed by NPs who are either retired or hold long time experience. As such, they acquired recognition and role legitimacy among patients as retired/senior government employees in the public system. They offer proximity-based services ―as they are familiar― in the community at an affordable price or even on credit bases for the worst-off. Additionally, their popularity could be associated with a mounting middle class, barely literate, acting in informal business, and relies on trust and easy access rather than on provider’s qualification. Despite their mere focus on curative care, NPs compete in the healthcare market, offering some specialized and invasive services. Furthermore, their steady development subsequently made a booking of appointments easier, allowing their patients to avoid unnecessary queuing as often witnessed in the public sector. Finally, concerning most females choosing FP and the absence of a dose-response association between education, or job status and FP preference (vs NFP), we argued this to be due to the blurring role of FP nurse in the hierarchy underscore by Das and Hammer [40]. Thus, in Burkina Faso, high and less educated, good jobholder and being unemployed, and females who are not among the well-off of the society, all together resorted to FP nurses' services.

Thirdly, insurance played an important role in choosing either MD-led FP over MD-led NFP for both severe and non-severe clinical conditions. Most holders possess a private or corporate-based scheme and are reimbursed at up to 80–100%. Such an association motivates the choice for the well-known MDs even for minor health conditions; besides, insurance companies may be more prompt to refund MDs' prescriptions more than other providers. People with insurance voluntarily take advantage to be seen in an FP facility by a famed MD general practitioner or a specialist (moonlighting or not), even for minor conditions. MD-led FP are more likely to be solicited because they are more easily bookable and their number overweight MD-led NFP. Service fees at MD-led NFP are comparatively very low [46], consequently often highly solicited and over-crowded.

Fourth, adjusted results showed that educational attainment was a predictor of opting for MD-led FP facilities. This appears to contradict Druss [47] who did not find any difference in education level between patients visiting MDs and NPs but the study did not distinguish public to private facilities. Three elements may justify our finding. First, educational attainment has been found to correlate with health literacy [48] and this may explain the awareness of highly educated in identifying the most appropriate provider category when a given case arises. Second, it is worth noting the parallel correlation of education and insurance coverage in the likelihood of choosing between MD-led FP and MD-led NFP facilities and between MD- and nurse-led facilities. From our data, the proportion of insured participants grows with the education level, and insurance appears to play a predictive role in making choices for MD-led FP facilities. Lastly, in the context of the study, highly educated, due to their occupation are quite fussy about waiting time and/or, amenities for which MD-led FP facilities are more cognizant of compared to other facilities.

## Limitations

The study embeds several limitations. The use of a cross-sectional design prevented us from examining the effect of continuity of care on the patient-provider relationship. Provider choice continuity is potentially an important covariate that needs further exploration in future studies. Another limitation concerns the symptom-based classification of the condition severity (severe versus non-severe). Misclassification could occur because participants may perceive some symptoms more than others or have a special focus on one or some during a disease episode. Thirdly, there are three largest MD-led NFP facilities in the city of Ouagadougou, and the gatekeeping mechanism suggests that patients—almost all—are being first attended by nurses and then, serious cases are referred to MDs. Because it was not possible to disentangle that, all their users were considered as MD-led NFP visits. This certainly may have minored or underestimated nurses' role and must be taken with caution. A number of studies including RCTs contended that up to 95% of commonly presented cases could be handled by nurses, unaided [49,50]. Fourth, considering the similarities of the private health sector in West Africa, the results may be insightful, however, may lack external validity due to the marked political, regulatory and socioeconomic differentials between states [51]. Fifth, because of the lack of a tight regulation, some doctors take advantage of meeting patients in nurse-led FP clinics. Thus, patients may have misclassified the qualification of the provider of their usual source of care, however, our study was unable to differentiate between RNs and NPs what may be less problematic. However, if present, misclassification would more frequently result in nurses being misclassified as doctors rather than doctors as nurses, but great efforts have been invested to tackle this. Asides from these limitations, several strengths were noted. Our results are strengthened by the strict application of probabilistic methods to map out households that could ensure a better internal validity. Our study is probably the first, to the best of our knowledge, to directly compare doctors and nurses in private-practice with emphasis on the consumer perspective. The findings may be of importance to encourage replication elsewhere in SSA countries witnessing similar dynamism in private healthcare sector development.

## Conclusion

This research indicates that the rising tide of private health systems in SSA urban areas contributes and competes in the healthcare market with the traditional public health sector. The study promotes the understanding of individuals’ healthcare-seeking behavior in nascent private healthcare. More light was shed on consumers' healthcare-seeking behavior and nurses as well as MDs' contribution to the private healthcare landscape, being FP or NFP. Findings show significant differences in insurance holding as well as higher education as main vectors of resorting to MD-led or nurse-led facilities regardless of the ownership or the severity of health condition. Besides higher education and insurance, holding a good job makes people favor FP- over NFP-led facilities. These unique, but progressive findings from a low-income country offer practical implications on how to strengthen each actor’s contribution in an evidence-informed policy era focuses on cost reduction, while increasing access and quality of care is highly critical. Additionally, this study provides knowledge, translatable to strategic health workforce policy development as well as potential role redistribution or clarification towards improving access to health care. These findings have the potential to light up related policies in other SSA countries, especially those within the Western Africa sub-region with similar healthcare systems that are working towards the attainment of SDGs.

## Supporting information

S1 Dataset (SAV)
